# Interleukin-28B genotype testing to determine response to the combination of pegylated-interferon and ribavirin for the treatment of hepatitis C virus

**DOI:** 10.1371/currents.RRN1207

**Published:** 2011-01-08

**Authors:** Christine M. Nguyen, Margaret Mendes, Shirley Tsunoda, Joseph D. Ma

**Affiliations:** ^*^VA San Diego Healthcare System and ^‡^UCSD Skaggs School of Pharmacy and Pharmaceutical Sciences

## Abstract

Hepatitis C virus (HCV) is a bloodborne infection that is one of the leading causes of liver disease. If left untreated, HCV can lead to cirrhosis, hepatocellular carcinoma, and death. The current standard of care for HCV is a combination of pegylated-interferon (peg-IFN) and ribavirin (RBV) in which the goal of treatment is to decrease complications and death due to HCV. HCV displays genetic polymorphism, where patients with HCV genotype 1 may have higher viral replication rates and are less likely to respond to treatment. These patients require a longer duration of treatment and a higher RBV dose. The interleukin (IL) 28B genotype test is associated with a sustained virologic response (SVR), defined as an undetectable HCV ribonucleic acid (RNA) upon completion of treatment and 24 weeks thereafter.

## **Clinical Scenario**

The IL28B genotype test can be used to predict response to peg-IFN and RBV in HCV genotype 1 patients. The test result indicates whether the patient has an IL28B CC, CT, or TT genotype. Patients who have the IL28B CC genotype are more likely to have a SVR with peg-IFN and RBV treatment, whereas patients who have the TT genotype are more likely to be nonresponders. This information can help clinicians and patients make informed decisions on how to best manage their HCV infection.  

## **Test Description**

The IL28B polymorphism test is a polymerase chain reaction (PCR) analysis of a single nucleotide polymorphism (SNP) (rs12979860 C/T) on chromosome 19q13.[1] The test requires either whole blood or buccal swab.

## **Public Health Importance**

Chronic liver disease is the tenth leading cause of death in the United States. The Center for Disease Control and Prevention (CDC) estimates that 40% of chronic liver disease is caused by HCV.[2] Treatment of HCV can reduce the risk of developing cirrhosis, hepatocellular carcinoma, and death. Treatment also prevents person-to-person transmission. However, SVR does not occur in all patients. Two clinical trials have showed that only 41% and 46% of patients with HCV genotype 1 achieved SVR with peg-IFN and RBV.[3] [4] In addition, many patients do not complete treatment due to adverse events that are associated with peg-IFN and RBV.[2] [5] Selecting appropriate and motivated patients to treat is important. The IL28B genotype test may aid healthcare providers and patients when making decisions on whether or not to start therapy in HCV genotype 1 patients.

## **Published Reviews, Recommendations and Guidelines** 

**Systematic evidence reviews**

**

None identified

**Recommendations by independent group**

**

None identified  

**Guidelines by professional groups**

**None identified 

## **Evidence Overview**

PubMed and Cochrane Review searches were conducted using a combination of the following keywords: IL28 polymorphism genotyping, hepatitis C virus, interferon, and ribavirin. The results were limited to English, and yielded a total of nine relevant articles.  

***Analytic Validity*** No information regarding the analytical validity of the IL28B genotype test was available in either the primary literature or on the LabCorp website.[6]

***Clinical Validity***

- Among patients who achieve SVR, the frequency of CC genotype in Caucasian, Hispanics, and African Americans are 69%, 56%, 48%, respectively.[7]
- Overall, the SVR rate is higher among patients who have the CC genotype ranging from 60% to 75%.[8] [9] As compared to patients with the TT genotype, Caucasian and Hispanic patients with the CC genotype have a two-fold likelihood in achieving SVR.[10] African American patients with the CC genotype have a three-fold likelihood of achieving SVR as compared to the TT genotype.[10]
- There is no difference in the prevalence of the various IL28B genotypes between patients infected with HCV and those who are co-infected with HIV.[9] [11] [12] However, a recent study showed that co-infected patients with the rs8099917 SNP G allele had a higher incidence of treatment failure with Peg-INF and RBV.[13] The current IL28B genotype test result does not account for any polymorphisms on rs8099917.
- One study reported the sensitivity, specificity, positive predictive value (PPV), and negative predictive value (NPV) of the CC genotype in predicting SVR in HCV patients who are IFN treatment naïve.[14] Sensitivity: 49.5%; specificity: 85.7%; PPV: 90.7%; NPV: 58.8%. Of note, the study included HCV patients regardless of their genotypes.

***Clinical Utility***

- Studies have shown a PPV of the IL28B polymorphism and SVR in HCV genotypes 1 and 4.[10] [14]
- The CC genotype is more common in HCV genotype 2 or 3 patients who achieve SVR.[15] However, the IL28B genotype test in these patients may not be necessary as they have a higher SVR rate as compared to HCV genotype 1 or 4 patients.[3] [4]
- Several SNPs have been studied in clinical trials, including rs12979860 and rs8099917. However the current IL28B genotype test only analyzes rs12979860.[14] [16] [17]
- In the absence of cost-effectiveness analysis, institutions should determine the cost-benefits of using the IL28B genotype test in HCV patients based on the available data and test costs.

## **Funding Information**

Christine M. Nguyen and Margaret Mendes are funded by the Veterans Affairs San Diego Healthcare System. Shirley Tsunoda and Joseph D. Ma are funded by the University of California, San Diego Skaggs School of Pharmacy and Pharmaceutical Sciences.  

## **Competing Interests**

## The authors have declared that no competing interests exist.
